# Experimental Study on Enhanced Oil Recovery of PPG/ASP Heterogeneous System after Polymer Flooding

**DOI:** 10.3390/gels9050427

**Published:** 2023-05-19

**Authors:** Yanfu Pi, Zailai Su, Ruibo Cao, Bo Li, Jinxin Liu, Xinyu Fan, Mingjia Zhao

**Affiliations:** 1 Key Laboratory of Enhanced Oil and Gas Recovery of Ministry of Education, Northeast Petroleum University, Daqing 163319, China; 2 Enhanced Oil Recovery Laboratory, Exploration and Development Research Institute, Daqing Oilfield Company Limited, Daqing 163712, China

**Keywords:** precrosslinked particle gel, post polymer flooding, heterogeneous system, profile control and flooding, enhanced oil recovery

## Abstract

Following the application of polymer flooding in Daqing Oilfield, the heterogeneity between different layers has intensified, resulting in the formation of more favorable seepage channels and cross-flow of displacement fluids. Consequently, the circulation efficiency has decreased, necessitating the exploration of methods to enhance oil recovery. This paper focuses on experimental research utilizing a newly developed precrosslinked particle gel (PPG) combined with alkali surfactant polymer (ASP) to create a heterogeneous composite system. This study aims to improve the efficiency of heterogeneous system flooding after polymer flooding. The addition of PPG particles enhances the viscoelasticity of the ASP system, reduces the interfacial tension between the heterogeneous system and crude oil, and provides excellent stability. The heterogeneous system has high resistance and residual resistance coefficients during the migration process in a long core model, achieving an improvement rate of up to 90.1% under the permeability ratio of 9 between high and low permeability layers. Employing heterogeneous system flooding after polymer flooding can increase oil recovery by 14.6%. Furthermore, the oil recovery rate of low permeability layers can reach 28.6%. The experimental results confirm that the application of PPG/ASP heterogeneous flooding after polymer flooding can effectively plug high-flow seepage channels and improve oil washing efficiency. These findings hold significant implications for further reservoir development after polymer flooding.

## 1. Introduction

Polymer flooding, currently the most widely used method for chemical enhanced oil recovery, has proven effective in improving the recovery rate [1,2]. In the case of Daqing Oilfield, the industrial application of polymer flooding was successfully implemented in 1996, achieving a recovery rate of 56%. However, certain challenges emerged during the development process as the scale of polymer flooding applications expanded. The challenges include limited recovery rate improvement, high polymer consumption, increased water content after polymer flooding, and excessively rapid production decline [3,4,5]. After polymer flooding, the heterogeneity of the reservoir becomes more pronounced compared to water flooding, making it more susceptible to water fingering and leading to the dispersion of the remaining oil. To address these issues, Daqing Oilfield progressed to tertiary (ASP) compound flooding technology, which significantly enhanced the recovery rate [6,7,8]. However, the ASP system failed to fundamentally solve the challenges caused by improving the mobility ratio as the oil displacement mechanism [9]. The long-term seepage channels resulted in a noticeable increase in the permeability of high permeability layers and a substantial reduction in the residual oil saturation. In contrast, the adsorption and retention of polymers and chemicals in low permeability layers led to decreased reservoir permeability [10], further exacerbating the heterogeneity between layers and impeding the efficient circulation of drive fluids.

Researchers have conducted extensive investigations on in-depth profile control of post-polymer flood reservoirs to address the inefficient circulation of displacement fluids. Seright studied the plugging effect of strong and weak gels in reservoirs with different permeabilities [11]. Wang Hongguan used in-situ polymerization to modify polyacrylamide and developed a cost-effective fluid diversion agent for deep profile control [12]. Zhao systematically analyzed a phenolic resin crosslinked nonionic polyacrylamide gel for in-depth profile control. [13]. Among the in-depth fluid diversion agents, precrosslinked particle gel (PPG) has garnered attention [14], with researchers utilizing its plugging effect to block high-flow channels and expand the volume of displacement fluids. Wang designed a heterogeneous composite system for the post-polymer flood reservoir in Shuanghe Oilfield and conducted experiments, including flooding agent optimization [15], thermal stability evaluation, and injection method optimization. The oil recovery rate can be improved by 27.8% after implementing the polymer flood. Bai [16] found that the concentration of crosslinking agents significantly influenced the strength and expansion performance of PPG, while the dosage of main agents and initiating agents had a lesser impact. Leng studied the mechanism and empirical model of particle gel transport and retention [17]. Sun studied the movement of elastic microspheres under shear stress [18], which can travel to larger pore channels with lower flow resistance, effectively forming plugs and diverting the fluid flow. Sang found that PPG effectively improves reservoir heterogeneity, with a more pronounced effect on increasing the recovery rate of low-permeability layers as the heterogeneity intensifies [19]. Shengli Oilfield studied the B-PPG heterogeneous compound flooding technology and conducted pilot tests for polymer and heterogeneous compound floodings, resulting in a 7.8% increase in recovery rate during the field application [20]. Similarly, relevant heterogeneous flooding experiments have also been conducted in Daqing and Zhongyuan Oilfields, showcasing significant improvements in oil recovery [21]. The development and application of PPG provide a novel approach to enhancing the recovery rate. In this study, the newly developed PPG from the Exploration and Development Research Institute of Daqing, characterized by high viscosity and elasticity, was used to form a heterogeneous compound system with alkali, surfactants, and polymers. Laboratory experiments were conducted to evaluate the performance of the heterogeneous system, including its profile control ability and transport behavior in long cores. In addition, the heterogeneous system was assessed for oil recovery using a three-pipe parallel model. This study provides a theoretical foundation for future field applications and holds great significance in extending the production life of aging oilfields.

## 2. Results and Discussion

### 2.1. Precrosslinked Particle Gel Swelling Ratio

The swelling capacity of PPG particles plays a crucial role in determining their compatibility and sealing effectiveness within the oil reservoir. PPG particles were dissolved in a 99.5% ethanol solution and produced water to assess this property. A comparison of particle sizes before and after the dissolution of PPG particles is presented in Table 1, revealing the swelling factor of PPG particles to be approximately 3.5 times, indicating that PPG particles possess excellent water absorption and swelling properties.

### 2.2. Performance Evaluation of PPG/ASP System

The viscoelasticity of the precrosslinked particle gel is a critical factor influencing the effectiveness of in-depth profile control. Key parameters for evaluating viscoelasticity included the storage modulus G’ (elastic modulus) and loss modulus G” (viscous modulus). The storage modulus serves as an indicator of the PPG’s strength, with a higher modulus value indicating a lower likelihood of deformation for PPG particles. On the other hand, the loss modulus represents the system’s viscosity. The results of viscoelasticity experiments using the heterogeneous system are shown in Table 2. Mixing PPG with ASP increased both the viscosity and storage modulus of the ASP system. Consequently, the incorporation of PPG particles increased the viscosity of the ASP system by 18%, resulting in a reduced polymer requirement. Since the PPG/ASP system is heterogeneous, PPG particles are suspended within the ASP system. The increase in viscosity not only reduced the flow ratio but also enhanced the system’s ability to carry PPG particles.

**Figure 1:**
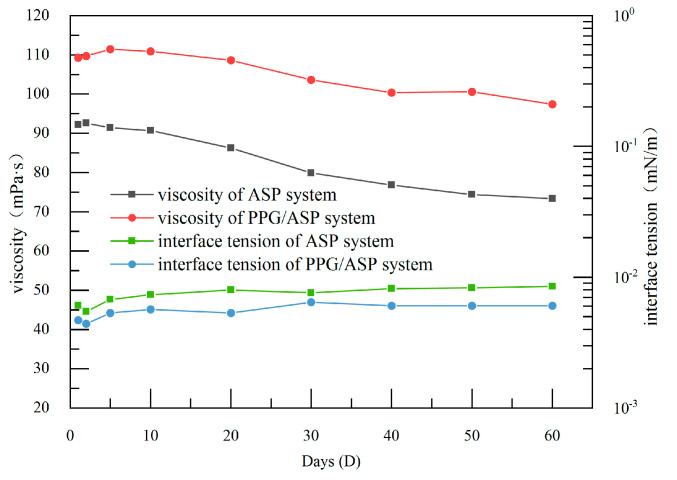
Time-dependent curve of viscosity and interfacial tension.

In practice, it takes several months for the oil displacement system to be injected into the well and extracted from the production well. During this period, it is crucial for the viscosity and interfacial tension of the oil displacement system to maintain a high level of stability. The results of the aging experiments on the viscosity and interfacial tension of both systems are depicted in Figure 1. After 60 days, the heterogeneous system has a viscosity retention rate of 89.3%, while the ASP system has a viscosity retention rate of 79.5%. The addition of PPG particles improves the system’s viscosity and viscosity retention rate. Compared to the ASP system, where the viscosity continues to decrease, the viscosity of the heterogeneous system remains stable after 30 days. The interfacial tension of the systems shows minimal changes over time, with the PPG/ASP system exhibiting slightly lower interfacial tension than the ASP system. From an emulsification standpoint, reducing interfacial tension is beneficial for improving crude oil recovery.

### 2.3. Production Profile Improvement Capability

**Figure 2:**
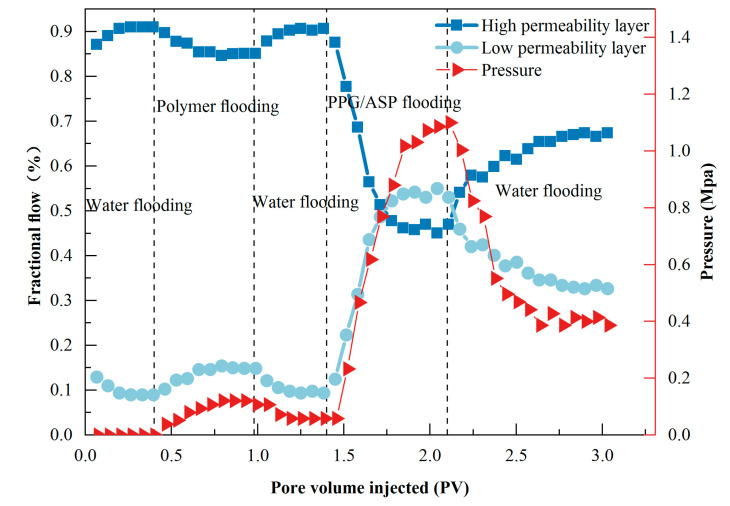
Flow rate and pressure curve of the double-tube parallel experiment.

Due to the heterogeneity of the formation, PPG particles exhibited notable selectivity towards pores with different permeability, resulting in a migration pattern characterized by “clogging-high, bypass-low” in heterogeneous systems. The production profile improvement ratio is commonly used to assess the effectiveness of selective flooding, indicating the degree of improvement in profiles across different permeability layers [22]. The experimental results are shown in Figure 2. 

During the water flooding stage, the flow rate ratio between high and low permeability layers was approximately 9:1, leading to significant channeling phenomena. Although the volume of the absorbed agent in the low permeability layer slightly increased during the polymer flooding stage, it decreased again during the subsequent water flooding stage. Analysis revealed that the improvement of profiles by the intermediate polymer flooding was limited and quickly lost its effectiveness. It was evident that increasing liquid phase viscosity alone could not effectively change the absorbed ratio across different layers. 

In the PPG/ASP heterogeneous system stage, the pressure increased sharply. The flow rate ratio of low permeability layers increased to 52.2%, resulting in a reversed imbibition water saturation profile and a profile improvement rate of 90.1%. PPG particles exhibited a tendency to block high-permeability layers while bypassing low-permeability layers. PPG particles accumulated and blocked large pores in high-permeability layers, forcing subsequent displacing fluids to enter smaller pores, thereby increasing flow resistance and inlet pressure. Since the permeability of the low-permeability layer was small, PPG particles could not pass through and instead formed a new pore structure at the inlet without affecting the ASP displacing fluid. 

During the subsequent water flooding process, the heterogeneous system was diluted by water, causing a significant decrease in system viscosity and subsequent flushing out of some PPG particles. As a result, the flow resistance of high-permeability layers decreased, resulting in reduced pressure. The flow rate ratio in high permeability layers increased to 66.3%, while the profile improvement rate remained stable at 77.6%. The experimental results demonstrated that the heterogeneous system could effectively block the high-permeability layer under the permeability ratio of 9 between high and low permeability layers, achieving a reversed imbibition water saturation profile and maintaining a good profile improvement ability during subsequent water flooding.

### 2.4. Seepage Law

Due to the complexity of PPG migration in ternary systems, flow experiments were conducted using a long core model to investigate the pressure characteristics of two oil displacement systems during the flow migration. The flow characteristics of the two oil displacement systems were analyzed by calculating the resistance coefficients *F_r_* and residual drag coefficients *F_rr_*. The magnitude of the *F_r_* value indicates the strength of the oil displacement and control of the interfacial flow. A higher *F_r_* value results in stronger flow control, with a larger area affected by the system expansion. In contrast, the *F_rr_* value reflects the ability to reduce the relative permeability of water. A higher *F_rr_* value implies a lesser likelihood of waterflood. The results of calculating the resistance and residual drag coefficients are presented in Table 3. 

Comparing the *F_r_* and *F_rr_* values of the two systems at three different positions along the long core, the heterogeneous system had significantly higher *F_r_* and *F_rr_* values, indicating its ability to effectively reduce the relative permeability of water and control the flow of the displacement, thereby expanding the affected volume. In the front 1/3 section of the core, both oil displacement systems achieved their maximum *F_r_* and *F_rr_* values. Therefore, the retention of PPG particles in the front 1/3 of the long core was the highest. Additionally, the heterogeneous system experienced shear forces within the core, leading to a corresponding decrease in system viscosity. These two factors jointly contributed to the maximum *F_r_* value observed in the front section of the core.

**Figure 3:**
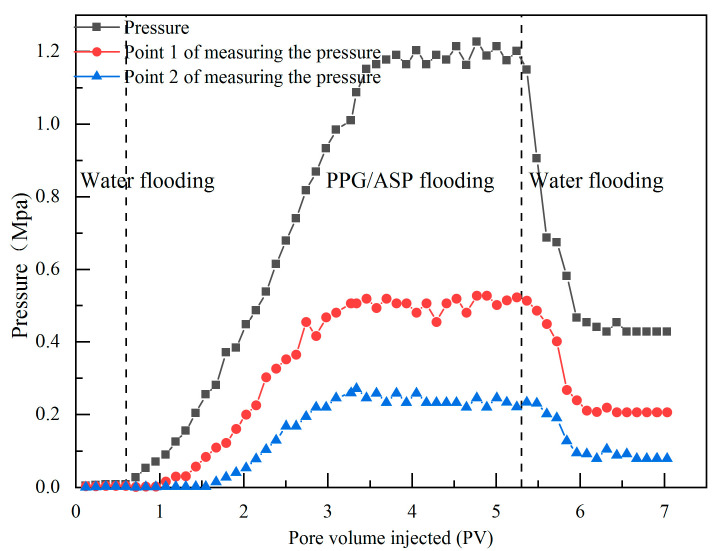
Pressure curve of PPG/ASP system.

The pressure curve of heterogeneous systems during the migration process in the long core is depicted in Figure 3. During the injection of heterogeneous systems, the injection pressure rapidly increased, surpassing that of the ASP system. The pressures at the three measuring points reached a certain level, then showed a fluctuating and stable pattern [23]. Analysis reveals that PPG particles enter the pore channels and accumulate at the pore throat, causing the subsequent fluid flow to redirect and leading to a significant increase in displacement pressure. Thus, a threshold pressure exists at the blocked pore throat [24]. When the pressure exceeds the threshold pressure at the accumulation point, PPG particles are squeezed, deformed, or even break through the pore throat, leading to a pressure drop at the throat. This process repeats continuously throughout the injection process, resulting in an unstable upward pressure fluctuation at various measurement points. 

After injecting 2.8 PV (pore volume) of heterogeneous systems, the injection pressure fluctuated around 1.17 MPa, indicating that the PPG particles inside the rock achieved a dynamic equilibrium state during the migration process. In the pressure rise stage at each measuring point, the pressure gradients were determined to be 3.45, 1.3, and 1.1 MPa/m, respectively. The results suggest that the migration process of PPG particles leads to their progressive size reduction and a gradual decrease in the blocking effect. During the injection of the PPG/ASP system, the pressure near the outlet became dynamically stable before the injection pressure reached a stable state. This stable state was propagated from the outlet to the inlet.

### 2.5. Evaluation of Oil Displacement Effect

A three-tube parallel model was utilized in oil displacement experiments to simulate the residual oil distribution in heterogeneous reservoirs after polymer flooding. This study aimed to examine the impact of the PPG/ASP and ASP flooding systems on the further recovery of residual oil. The results of the oil displacement experiments are presented in Table 4. 

During the water flooding and polymer flooding stages, the majority of oil production originated from high-permeability and medium-permeability layers, while low-permeability layers contributed minimally. After polymer flooding, the recovery rate of high-permeability and medium-permeability layers was 61.7% and 52.6%, respectively, and the overall recovery rate was 44.6%. Compared to the ASP flooding stage, the heterogeneous flooding stage led to an increase of 5.5% in the total oil recovery due to enhanced oil recovery from low–medium permeability layers. The recovery rate of low-permeability layers was 28.6%, and the overall oil recovery was 60.4%. 

**Figure 4:**
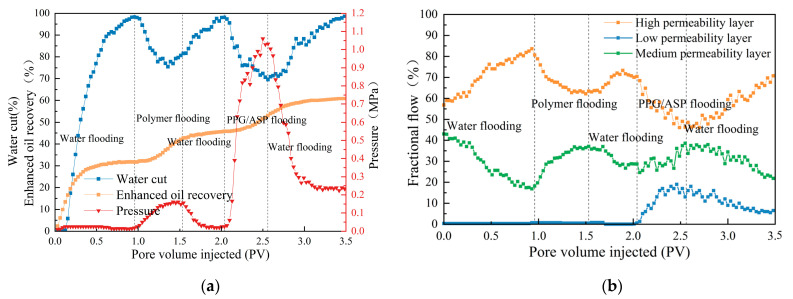
Production curves during PPG/ASP flooding: (**a**) Pressure, water cut, and oil recovery as a function of the injected pore volume of PPG/ASP flooding. (**b**) Displacement flow rate curves of the heterogeneous system.

To further analyze the effects of the heterogeneous system on different layers, the production curves of the heterogeneous system and the fractional flows of each layer are plotted in Figure 4. During heterogeneous flooding, the pressure increased rapidly, resulting in a 30% reduction in total water content. The fractional flow rate of the high-permeability layer exhibited a fluctuating decline, with a reduced rate of up to 25.2%. In contrast, the fractional flow rate of the medium and low permeability layers showed a fluctuating increase, with an increased rate of 38.5% and 20.2%, respectively. These observations indicate that the breakthrough occurred when the displacement fluid successfully entered the low-permeability layer, resulting in a practical displacement effect [25]. Notably, the oil production from the low-permeability layer accounted for approximately half of the total oil production during the heterogeneous dominant process, indicating that the ASP/PPG heterogeneous system could effectively enhance oil production in the low-permeability layer. 

In the subsequent water flooding stage, PPG particles were carried away by water, causing a decrease in the injection pressure. As a result, the liquid-sweeping ratio of the high-permeability layer recovered to some degree. However, a high residual resistance coefficient still existed, making it challenging for the subsequent water flooding to break through. Thus, the water flooding time during the heterogeneous stage significantly improved compared to the polymer-flooding stage. In conclusion, the ASP–PPG heterogeneous system can improve the displacement efficiency of the heterogeneous reservoir after polymer flooding, thereby increasing the recovery rate of the reservoir. The system effectively utilized the effect of PPG particles on blocking the permeability channels through an “aggregating-blocking-deforming-penetrating” process, leading to improved sweep efficiency and redirecting the follow-up displacement fluid to the unreached area and low-permeability layer [26]. The synergistic and enhancing effects between PPG and ASP prove the advantages of the system in enhancing oil recovery.

## 3. Conclusions

The objective of this study was to address the challenges posed by increased heterogeneity and the presence of high flow paths in reservoirs after polymer flooding in Daqing oilfield. The displacement experiments were conducted to investigate the performance of the PPG/ASP heterogeneous system on enhancing oil recovery after polymer flooding. The main conclusions and recommendations are the following:

(1) PPG particles possess excellent water absorption and swelling properties, with a swelling ratio of 3.47, high viscosity, and elasticity. The PPG/ASP system exhibits ultra-low interfacial tension and high viscosity, with a viscosity retention rate of 89.3% even after 60 days.

(2) The PPG/ASP system has excellent flow performance and profile control capabilities. It effectively reached the deeper regions of the core to control liquid flow. High resistance and residual resistance coefficients were obtained during the migration process. The profile improvement rate reached 90.1% under the permeability ratio of 9 between high and low permeability layers.

(3) The PPG/ASP heterogeneous system further improved oil recovery by 14.6% after polymer flooding. The oil recovery of high, medium, and low layers after polymer flooding was increased by 6.2%, 13.4%, and 28.6%, respectively, and the ultimate oil recovery rate was 60.4%.

(4) In practical applications, injection pressure becomes critical when using heterogeneous flooding technology. It is essential to ensure that the actual injection pressure is maintained below the fracture pressure of the formation. Effectively managing the injection of heterogeneous systems within the permissible pressure range is key to successfully applying heterogeneous system flooding in oilfields.

## 4. Materials and Methods

### 4.1. Experimental Materials

This study utilized specific chemical reagents and materials, which are listed below:

- Polymer: Polyacrylamide (HPAM) with two molecular weights, which are 25 million and 14 million. The polymer had an effective content of 90% and a hydrolysis degree of 30%, produced by PetroChina Daqing Petrochemical Company (Daqing, China).
- Alkali: Anhydrous Na_2_CO_3_ with a purity of 99%.
- Surfactant: Petroleum sulfonate with a mass concentration of 20%, produced by Daqing Wantong Chemical Co., Ltd (Daqing, China).
- Precrosslinked particle gel (PPG): The PPG used in this study was developed by the Exploration and Development Research Institute of Daqing Oilfield (Daqing, China).
- Experimental water: The water had a mineralization degree of 6778 mg/L and a pH of 8.5. The specific composition and concentration of experimental water are detailed in Table 5.
- Other material: Produced water was obtained from the Daqing No.1 factory (Daqing, China). Dehydrated crude oil was achieved from the Daqing oil field production plant (Daqing, China). Aviation kerosene. The simulated oil used in the experiment was prepared by blending dehydrated crude oil and kerosene with a viscosity of 9.8 mPa·s at 45 °C.

According to the data obtained from the core samples retrieved from a typical block in Daqing Oilfield [14], the permeability of the high, medium, and low reservoir layers was approximately 4000, 2000, and 500 (×10^−3^ μm^2^), respectively. The ratio of reservoir thickness was approximately 18:45:20. The core models were designed based on actual reservoir conditions and cast using epoxy resin. The specific parameters of the cores used in the experiments are shown in Table 6. The mass fraction of alkali and surfactant used in this study was 1.2% and 0.3%, respectively [7]. The concentration of polymer (25 million) used in experiments was 1200 mg/L, and the PPG concentration was 500 mg/L.

### 4.2. PPG Particle Swelling Experiment

A certain amount of PPG powder was dissolved in a 99.5% concentration ethanol solution and produced water to prepare a test PPG solution. The prepared solution had a concentration of 500 mg/L. After settling the solution at 45 °C for 1 h, the PPG particle size distribution was determined using a MICROTRAC S3500 laser particle analyzer. The initial PPG particle size was denoted as *M_d_*_1,_ and the soaked PPG particle size was referred to as *M_d_*_2_. The swelling ratio (*S*) was calculated using the median particle size of *M_d_*_1_ and *M_d_*_2_.

(1) $$S=\frac{M_{d2}}{M_{d1}}\;$$

### 4.3. System Performance Measurement

The ASP system and PPG/ASP system were prepared using clean water, then diluted with produced water. The HAKKE MARS rheometer was used to determine the elastic modulus and viscous modulus of each system at a fixed stress and frequency under the temperature of 45 °C, and then the two systems were aged at 45 °C. The viscosity of the systems was measured at regular intervals using a BROOKFIELD digital viscometer to obtain the variation function of viscosity with time. During the aging process, the Texas-500C rotary drop interfacial tension meter was used to measure the interfacial tension between the system and crude oil under 45 °C and 4500 r/min.

### 4.4. Profile Control Evaluation Experiment

**Figure 5:**
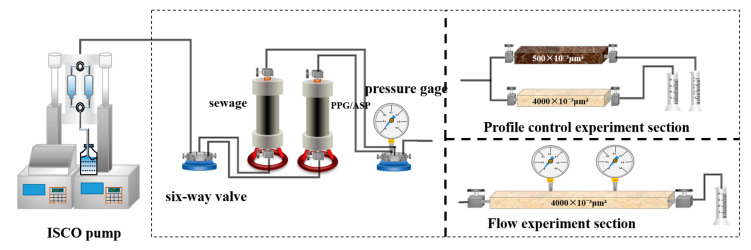
Profile control experiment and flow experiment setup.

The experiment utilized a dual-tube parallel model to simulate a high permeability layer and a low permeability layer with permeability of 4000 mD and 500 mD, respectively. The characteristic of adjusting the imbibition water saturation profile of the reservoir by the PPG/ASP system was studied by calculating the profile improvement rate. The experimental setup is shown in Figure 5. The experiment procedure is presented as follows: (1) The high and low permeability cores were vacuumed, saturated with formation water, and aged in formation water under the experimental temperature of 45 °C for 24 h; (2) The injection rate was set to 0.7 mL/min, and the oil was displaced at a relatively stable pressure; (3) 0.7 PV of the PPG/ASP oil displacement system was injected; (4) The oil displacement continued until the pressure reached a relatively stable state. The pressure at the inlet and flow rates of each layer were recorded before and after the PPG/ASP system flooding and at the end of the experiment. The profile improvement rate (*f*) was calculated using the following formula:

(2) $$f=\left(1-\frac{Q_{H2}Q_{L1}}{Q_{H1}Q_{L2}}\right)\times 100\%\;$$

where *f* is the profile improvement rate; *Q_H_*_1_ and *Q_H_*_2_ are the pre and post-adjustment flow rates of the high permeability tube, respectively; *Q_L_*_1_ and *Q_L_*_2_ are the pre and post-adjustment flow rates of the low permeability tube, respectively.

### 4.5. Flow Experiment

The flow experiment utilizes a 60 cm artificial cemented rock core model with parameters provided in Table 2. Two pressure measurement points are positioned at distances of 20 cm and 40 cm from the injection end. The schematic of the experimental setup is shown in Figure 5. The experiment follows the procedure: (1) The rock core model is vacuumed and then saturated with formation water, followed by a 24-h settling period at an experimental temperature of 45 °C; (2) Produced water is injected with an injection rate of 0.7 mL/min until pressure stabilizes; (3) Two oil-displacing systems (ASP system and PPG/ASP system) are individually injected into cores until pressure stabilizes; (4) Produced water is then displaced until the pressure reaches a relatively stable state, and the pressure values of each section are recorded to conclude the experiment. The friction factor (*F_r_*) and residual friction factor (*F_rr_*) are calculated using the following equations:

(3) $$F_{r}=\frac{\Delta P_{p}}{\Delta P_{w1}}\;$$

(4) $$F_{rr}=\frac{\Delta P_{w2}}{\Delta P_{w1}}\;$$

where *F_r_* refers to the resistance coefficient; *F_rr_* represents the residual resistance coefficient; ∆*P_p_* denotes the pressure difference during the adjustment and flooding stage; ∆*P_w_*_1_ is the pressure difference during the water flooding stage before profile control; ∆*P_w_*_2_ denotes the pressure difference during the water flooding stage after profile control.

### 4.6. Oil Displacement Experiment

**Figure 6:**
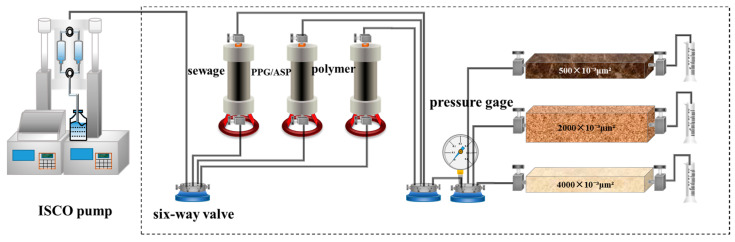
Connection diagram of oil displacement test device.

The oil displacement experiment involves a three-pipe parallel model with varying thicknesses of high, medium, and low permeability layers. The experimental setup is shown in Figure 6. The procedure of displacement experiments is presented as the following steps: (1) Evacuate the cores, saturate them with formation water, saturate them with oil, and age the three core models for 48 h; (2) Inject produced water at a rate of 0.7 mL/min and displace the oil until the water content of outlet reaches 98%; (3) Inject a 0.57 PV polymer solution (with a molecular weight of 12 million and a concentration of 1000 mg/L) and continue displacing the oil with produced water until the water content at outlet reaches 98%; (4) Inject 0.5 PV of each oil displacement system (ASP system and PPG/ASP system), and continue displacing the oil with produced water until the outlet water content reaches 98%. Record the pressure and the amount of produced oil and water by each layer and conclude the experiment.

## Figures and Tables

**Table 1:** Particle size distribution of PPG before and after dissolution and expansion.

Initial Particle Size/μm	Particle Size after Swelling/μm			Swelling Ratio/S
D_50_	D_10_	D_50_	D_90_	3.47
215.8	470.5	746.3	906.3	

**Table 2:** Comparison of viscoelastic and interfacial properties for different types of systems.

System	Storage Modulus G’/Pa	Viscous Modulus G”/Pa	Viscosity/mPa·s	Interfacial Tension/mN·m^−1^
ASP	0.343	0.589	92.2	6.06 × 10^−3^
PPG/ASP	0.782	0.968	109.2	4.69 × 10^−3^

**Table 3:** Resistance coefficient and residual resistance coefficient at different positions of the rock core.

Category		First 1/3 Section	Middle 1/3 Section	Last 1/3 Section
The ASP System	*F_r_*	140.0	48.3	32.0
	*F_rr_*	35.3	29.6	12.7
The PPG/ASP System	*F_r_*	232.1	104.4	88.0
	*F_rr_*	82.3	45.2	32.6

**Table 4:** Results of oil displacement experiments using various systems in heterogeneous reservoirs.

Layer		Initial Oil Saturation	Enhanced Oil Recovery (%)				
			Water Flooding	Polymer Flooding	Polymer Flooding Increment	PPG/ASP Flooding	PPG/ASP Flooding Increment
The ASP System	High	72.9	44.8	61.7	16.9	68.8	7.1
	Medium	72.4	32.2	52.6	20.4	62.5	9.9
	Low	71.7	2.7	4.3	1.6	7.8	3.5
	Total	72.6	30.9	44.6	13.7	53.7	9.1
The PPG/ASP System	High	73.1	45.2	62.2	17.0	68.4	6.2
	Medium	72.6	33.2	53.3	20.1	66.7	13.4
	Low	71.8	3.1	4.8	1.7	33.4	28.6
	Total	72.8	31.7	45.8	14.1	60.4	14.6

**Table 5:** Composition and concentration of various reagents in simulated formation water.

Reagent	NaHCO_3_	NaCl	KCl	MgSO_4_	Na_2_SO_4_	CaCl_2_
Concentration (mg/L)	3866	2545	28	88	161	90

**Table 6:** Core parameters of physical experiment models.

Type		Length × Width × Thickness (cm)	Permeability (×10^−3^ µm^2^)	Porosity (%)
Flow experimental models		60.0 × 4.5 × 4.5	3924	29.7
Profile control experimental model	High permeability	30.0 × 4.5 × 4.5	4067	30.2
	Low permeability	30.0 × 4.5 × 4.5	516	22.7
Oil displacement experimental model	High permeability	30.0 × 1.8 × 4.5	4055	30.1
	Medium permeability	30.0 × 4.5 × 4.5	2034	26.7
	Low permeability	30.0 × 2.0 × 4.5	523	22.8

## Data Availability

Data available on request due to restrictions e.g., privacy or ethical. The data presented in this study are available on request from the corresponding author. The data are not publicly available due to company requirements.
